# Editorial: Advances in protein-based biomaterials for tissue engineering

**DOI:** 10.3389/fbioe.2022.1022733

**Published:** 2022-09-08

**Authors:** Lei Nie, Ruixia Hou, Amin Shavandi

**Affiliations:** ^1^ College of Life Sciences, Xinyang Normal University, Xinyang, China; ^2^ Medical School of Ningbo Unversity, Ningbo, China; ^3^ École polytechnique de Bruxelles, 3BIO-BioMatter, Université libre de Bruxelles (ULB), Brussels, Belgium

**Keywords:** protein, tissue engineering, hydrogels, regeneration, biomaterials

Tissue engineering and regenerative medicine, via combining multidisciplinary sciences, including materials, engineering, biology, chemistry, and physics, aim to generate tissues and organs substitute for biomedical applications. Due to the excellent biocompatibility, low toxicity, immune reactivity, and low bacterial adherence, protein-based biomaterials (e.g. hydrogels) have been designed and studied for tissue engineering applications. Through selecting different biomaterials, crosslinking methods, and fabrication strategies, the physiochemical and biochemical properties of biomaterials could be designed and regulated, and cells could be encapsulated homogeneously to provide a 3D microenvironment similar to the native extracellular matrix (ECM). Thus, the design, preparation, and evaluation of biomaterials for tissue engineering still need to be further enhanced for future clinical applications, such as multi-functional properties, improved biocompatibility, cell generation efficiency, and so on. Many researchers have been interested in protein-based biomaterials with improved biocompatibility using effective strategies in several biomedical applications, including wound healing, cardiovascular stents, etc.

In this Research Topic, we report a number of interesting studies written by 35 authors, related to protein-based biomaterials with functional properties for tissue engineering applications. Due to the superior antibacterial properties and skin adhesiveness, polyzwitterionic hydrogels have been extensively studied for wound healing dressings. Lan et al. have designed a biocompatible composite polyzwitterionic hydrogel using quaternized chitosan methacrylate (QCSMA) and gelatin methacrylate (GelMA) as bioactive across-linkers, and the optimized hydrogel had high stretchability, low swelling, high bioactivity, strong skin adhesiveness, and antibacterial effect, with potential as a wound dressing for acute wounds. *Bombyx mori* silk fibroin (SF) has been manufactured in the forms of scaffolds, hydrogels, films, sponges and biomedical devices. The preliminary dissolution of native SF usually requires solvents such as formic acid (FA). Biagiotti et al. have investigated the type and strength of the interaction between FA and SF, and used the SILKBridge^®^ nerve conduit and confirmed the amount of FA left on the electrospun SF mats did not raise toxicological concerns for human health. Tissue engineering is also commonly used in the treatment of cardiovascular diseases, such as vascular interventional technique (VIT). Li et al. have developed a surfur-mediated polycarbonate polyurethane (PCU-SS) as a cardiovascular stent to mimic the catalyzing ability of glutathione peroxidase (GPx) on nitric oxide (NO) in the human body. In another research, Hu et al. have prepared a biomimetic dopamine-Zn^2+^ (DA-Zn^2+^) coating on the titanium surface using a one-step metal-catecholamine assembly strategy. Due to the continuous release of Zn^2+^ from DA-Zn^2+^ coating, the coated substrate displayed excellent hemocompatibility, antibacterial activity, and enhanced endothelial cell (EC) adhesion and proliferation. The method reported by Hu et al. can be applied to other cardiovascular implants for cardiovascular tissue engineering applications. Tissue engineering techniques could deliver specific types of cells to injured tissues or organs to restore tissue and organ function. The induced pluripotent stem cells (iPSCs) are generated and suggested as an alternative for the embryonic stem cells (ECSs), which raised ethical issues. Son et al. have reported a method using Lin28-30Kc19 fusion protein to improve the iPSCs generation efficiency.

Tissue engineering could overcome the conventional method of tissue regeneration and healing, such as the auto-graft method, which is mainly dependent on the availability of donor tissues. Therefore, the main aim of this Research Topic is to underpin the importance of biomaterials and cells in tissue engineering for biomedical applications. For instance, Lan et al. have shown a novel hydrogel as wound dressing, Li et al. have introduced a cardiovascular stent, Hu et al. have demonstrated a strategy to improve the biocompatibility of cardiovascular implants, and Biagiotti et al. have evaluated the toxicity of the electrospun SF, while Son et al. have constructed a method to improve the generation efficiency of iPSCs. We believe that the encouraging strategies and approaches could advance the tissue engineering techniques for future clinical applications. We sincerely hope you will enjoy reading all the papers in the special edition.

